# Pathways Linking Socioeconomic Circumstances to Childhood Dental Caries: The Mediating Role of Parenting and Oral Health Behaviours Before and After Childsmile Implementation

**DOI:** 10.1111/cdoe.70062

**Published:** 2026-03-09

**Authors:** Kyle Cousins, Mariél de Aquino Goulart, Paul Bradshaw, David I Conway, Andrea Sherriff

**Affiliations:** ^1^ School of Medicine, Dentistry, and Nursing, College of Medical, Veterinary and Life Sciences University of Glasgow Glasgow UK; ^2^ Scottish Centre for Social Research Edinburgh UK

**Keywords:** child health, dental caries, epidemiology, longitudinal, medical record linkage, socioeconomic factors

## Abstract

**Objectives:**

To determine the extent that parenting styles and oral health behaviours mediate the relationship between socioeconomic circumstances (SEC) and childhood dental caries experience: pre−/post‐national roll‐out in 2011 of Childsmile, the child oral health improvement programme for Scotland.

**Methods:**

A longitudinal design linked 2798 participants from Birth Cohort‐1 (following from age 10 months in 2005/06 to age 5 years in 2009/10) and 3015 from Birth Cohort‐2 (following from age 10 months in 2011 to age 5 years in 2015) of the Growing Up in Scotland study with caries experience at age five from the National Dental Inspection Programme. Two Structural Equation Models (Birth Cohort‐1 and 2) tested an a priori framework to explain the social gradient (SEC: Income Poverty, Area‐based Deprivation, Household Education, Household Employment) in caries experience, considering proximal behaviours (toothbrushing, cariogenic diet, regular dental attendance) before introducing validated parenting styles (Parental Responsiveness/Demandingness).

**Results:**

Models for both the pre−/post‐Childsmile cohorts illustrated similar pathways. Dental attendance and parenting had no direct effect on caries experience. Responsiveness partially mediated the SEC‐toothbrushing relationship, while demandingness partially mediated the SEC‐diet relationship. Cariogenic diet directly increased caries experience prevalence (*β* = 0.33/0.24), while increased toothbrushing persistently decreased caries (*β* = −0.18/−0.13). Total mediated effect of SEC decreased from 58.8% to 39.1%, with the largest relative decrease being directly via toothbrushing (9.5%/4.0%). The effect mediated through parenting pathways remained minimal (4.8%/3.5%).

**Conclusions:**

Pathways between SEC and childhood caries were unchanged after Childsmile roll‐out, with minimal effects from parenting styles. The diminishing SEC effects via toothbrushing may be positively attributed to Childsmile interventions. The persistent unexplained effect of SEC on caries experience highlights the need for equity‐focused structural approaches that address broader socioeconomic inequalities.

## 1 Introduction

Childsmile, the national child oral health improvement programme in Scotland, was introduced to improve overall oral health and reduce inequalities by addressing wider determinants beyond individual behaviours. Recognising the limitations of traditional oral health education, Childsmile was designed as a preventive, population‐level intervention, integrating oral health promotion within broader public health strategies, combining targeted and universal components across education, community and dental practice settings [1, 2]. Piloted in 2006 and rolled out nationally by 2011, Childsmile is subject to constant monitoring and evaluation of processes and outcomes.

The association between socioeconomic circumstances (SEC) and childhood dental caries is well‐established [3], but the mechanisms underlying this social gradient remain complex and multifactorial. SEC influence health through a combination of material, psychosocial and behavioural pathways, as outlined in the social determinants of health framework. Material pathways involve access to tangible resources such as nutritious food, oral hygiene products and preventive dental care. Psychosocial pathways could involve stress, parenting practices and the emotional environment shaped by SEC. Behavioural pathways encompass individual health‐related actions, such as toothbrushing and dietary habits. These mechanisms are interrelated; for example, material deprivation may constrain behavioural choices or exacerbate psychosocial stressors [4].

SEC is a multidimensional construct encompassing income, housing conditions, education and employment status, each reflecting different aspects of socioeconomic position and having both individual and area‐based dimensions [5]. For instance, household income affects access to nutritious food, while parental education shapes health literacy and caregiving practices [5]; notably, education is systemically intertwined with income and employment [6]. In Scotland, research often relies on an area‐based measure, the Scottish Index of Multiple Deprivation (SIMD) [7], which, while useful and cost‐effective, may not fully capture individual socioeconomic experiences [8].

Parenting styles, defined as the emotional and behavioural environment in which children are raised, represent a plausible but underexplored mediator between SEC and oral health. Grounded in Baumrind's framework, parenting styles—authoritative (high responsiveness, high demandingness), authoritarian (low responsiveness, high demandingness) and permissive (high responsiveness, low demandingness)—may influence children's oral health behaviours such as diet and oral hygiene, though evidence remains mixed [9, 10]. Two recent systematic reviews illustrate this ambiguity: one found consistent associations between parenting styles and children's oral health in 10 studies, whereas another only found significant relationships in five of nine studies, mostly linking authoritative parenting to lower caries risk and permissive parenting to higher risk. The dimensions of responsiveness and demandingness are considered relatively stable traits during early childhood.

This study draws on two birth cohorts from the Growing Up in Scotland (GUS) study—one born before Childsmile implementation (2004/05) and one during early roll‐out (2010/11)—to examine pathways linking SEC to childhood caries experience. It assesses the mediating roles of parenting styles and oral health behaviours, and whether these mechanisms differ before and after programme introduction. This work contributes evidence on how early Childsmile may have impacted on the processes underpinning oral health inequalities from early life.

The conceptual model underpinning this analysis is theoretically grounded in Bronfenbrenner's ecological systems theory [11], alongside a broader social determinants of health framework [12]. It aligns with the theory of multilevel influences put forward in conceptual models of child oral health [13]. The theoretical model is posited from previous findings that SEC are associated with parenting styles [14], and in turn parenting styles (responsiveness, demandingness) are associated with oral health behaviours (cariogenic diet, toothbrushing, regular attendance at dentist) and caries experience prevalence [9, 10].

## 2 Methods

This study leverages a natural experiment by analysing two cohorts of the GUS study. Structural equation modelling (SEM) was used to evaluate direct and indirect effects within a theoretically grounded life‐course framework, incorporating latent variables to reduce measurement error.

### 2.1 Theoretical Framework

**FIGURE 1:**
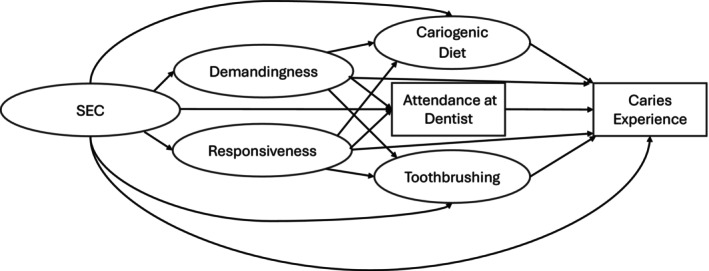
Theoretical model from Socioeconomic Circumstances to Caries Experience at age 5; through parenting styles and oral health behaviours. Freq, frequency; SEC, socioeconomic circumstances. Circular nodes: Latent variables; Rectangular nodes: Observed variables. Toothbrushing is observed, not a latent variable for BC2.

The pathways mentioned in the introduction and the theoretical model are shown in a Directed Acyclic Graph (Figure 1). The step‐by‐step algorithm for testing this model is detailed in Data S1.

### 2.2 Data Sources and Cohorts

This study used a longitudinal data linkage design combining two nationally representative GUS birth cohorts with dental inspection data from the National Dental Inspection Programme (NDIP) and National Health Service (NHS Scotland) primary dental care attendance records from the Management Information & Dental Accounting System (MIDAS).

- Birth Cohort 1 (BC1): 5217 children born in 2004/05, with data from sweep 2 (age 2) to sweep 5 (age 5)—pre‐Childsmile cohort.
- Birth Cohort 2 (BC2): 6127 children born in 2010/11, with data from sweep 1 (age 10 months) to sweep 3 (age 5)—immediately after early Childsmile roll‐out.

NDIP includes an annual census of caries experience on all Primary 1 school‐year (approximately 5 years old) children attending local authority schools.

GUS questionnaires were administered to participants by trained interviewers face‐to‐face using a computer‐assisted format [15].

Analytic samples were derived through progressive inclusion, carrying participants forward only if they: (i) provided consent for data linkage and were successfully linked; (ii) had data from all GUS sweeps used in the model; (iii) had a valid NDIP examination at age five; finally, those without complete‐case requirements were excluded (see Figure S1).

Individual participant‐level data linkage was performed securely by Public Health Scotland's eDRIS service, using probabilistic matching via the Community Health Index (CHI) number, where all analysis is conducted in the secure National Safe Haven. Ethical approval was obtained from the University of Glasgow MVLS College and Public Benefit and Privacy Panels (ref: 200170146).

### 2.3 Variables and Measures

All variables except primary care dental attendance and caries experience were collected from the GUS cohort surveys.

#### 2.3.1 Outcome

Caries experience (yes/no) at age 5 was derived from NDIP records. The dental inspection involves a simple assessment of the mouth and teeth of each child undertaken by trained and standardised dental teams within primary schools, recording obvious dental caries experience (decay into dentine).

#### 2.3.2 Exposure

SEC latent variable was constructed from four indicators (age 2/3): household income poverty defined as equivalised household income below 60% of national median income (annual median income at BC1 = £19 604 and at BC2 = £22 800), area‐level socioeconomic deprivation (SIMD), highest household education and parental occupational class (NS‐SEC) [16, 17, 18, 19]. Further description of these variables is in Data S1.

#### 2.3.3 Mediators

##### 2.3.3.1 Parenting Styles

Two latent constructs were created—Responsiveness and Demandingness—using items from the Pianta Child–Parent Relationship Scale, the Parenting Practices Questionnaire (PPQ) and GUS‐specific data on rules, routines, chaos and discipline [20, 21, 22]. Responsiveness generally reflects parental warmth and attentiveness, while Demandingness reflects the extent to which parents provide structure and set expectations. A full description of variables, coding and time points is included in S2.

#### 2.3.4 Oral Health Behaviours (Three Variables—Cariogenic Diet, Toothbrushing and Regular Dental Attendance)

##### 2.3.4.1 Cariogenic Diet (BC1: Age 2 and BC2: Age 3/5, Latent)

There were 29 possible diet variables in BC1 and 18 in BC2 (Table S3); many were mutually correlated and would not sit independently in a model; therefore, a process of data reduction was carried out ending with a latent class variable for diet. Variables with a non‐significant effect on the final latent variable or a factor loading < 0.4 were removed. Sweet consumption, sugar sweetened beverage (SSB) consumption and crisp consumption were used. The frequency of intake of each is assessed on an eight‐point scale from ‘never’ to ‘more than once a day’.

##### 2.3.4.2 Toothbrushing (BC1: Age 2 and BC2: Age 3, Latent in BC1)

For both cohorts, caregivers were asked how often a toothbrush was used to clean the child's teeth, with the following response options: Less than once a day; Once a day; More than once a day. For BC1, the latent variable is based on a combination of two questions:

- The frequency of toothbrushing (as described above).
- Whether toothpaste was used during toothbrushing, with a simple yes/no response.

For BC2, the observed variable is based on just the frequency of toothbrushing. However, for BC2 this question was changed to include ‘with fluoride toothpaste’.

##### 2.3.4.3 Regular Attendance at Dentist (Yes/no)

Defined as attending the dentist at least once a year for four out of 5 years from birth up to the child's 5th birthday from the MIDAS.

#### 2.3.5 Covariates

Age at NDIP Exam from NDIP databases and sex from GUS.

### 2.4 Statistical Analysis

#### 2.4.1 Preliminary Analysis

Univariable associations between all early life factors within GUS and caries experience were assessed using modified Poisson regression adjusted for age and sex. Predictive performance was evaluated via Receiver Operating Characteristic (ROC) curves (Tables S1 and S2). This was to help guide variable inclusion in final latent variables within model.

Survey weights are used throughout this analysis. The weights are developed using a model‐based weighting technique where response behaviour is modelled using data from previous sweeps detailed further in GUS User Guides [15].

#### 2.4.2 Structural Equation Modelling

SEM was conducted using the Lavaan package (version 0.6–19; Rossel, Zandberg, Albrich and Huttner [23]) in R Studio (version 2025.05.0). Given the categorical and ordinal nature of much of the data, models were estimated using the WLSMV estimator (Weighted Least Squares Mean and Variance adjusted), which provides robust standard errors and adjusted chi‐square statistics, ensuring valid inference under conditions of non‐normality and non‐continuous data. *p*‐values for individual pathways were derived from Wald *z*‐statistics, computed using asymptotically robust standard errors. Model specification followed an iterative process: non‐significant paths (*p* > 0.05) were removed sequentially, except where theoretical or empirical evidence indicated the variable's importance in the broader model structure.

Model fit was assessed using the chi‐squared statistic (χ^2^), Comparative Fit Index (CFI > 0.95), Root Mean Square Error of Approximation (RMSEA < 0.06) and Standardised Root Mean Square Residual (SRMR < 0.08). Where needed, modification indices guided the inclusion of theoretically justifiable residual covariances to improve fit and model stability.

Latent variables were used to capture shared variance among correlated observed indicators, reducing measurement error and providing more precise estimates of theoretical constructs. Confirmatory factor analysis supported the latent structure, with most factor loadings ≥ 0.4 and *p*‐values < 0.01 [24], except for ‘Toothpaste used (BC1)’ which had *p* = 0.08.

## 3 Results

Analytic samples were derived sequentially from the GUS birth cohorts (Figure S1). For BC1, 5217 respondents were recorded; of these, 3665 provided consent and were successfully linked. Among the linked cases, 3275 had data for all sweeps, and 2893 had a valid NDIP exam. The BC1 analytical sample included 2798 participants (96.7% of those with NDIP). For BC2, 6127 respondents were recorded; 5599 consented and were successfully linked. Of these, 3975 had data for all sweeps, and 3442 had a valid NDIP exam. The BC2 analytical sample had 3015 participants (87.6% of those with NDIP).

Missingness was < 5% for most variables except Poverty Status in BC2 (12.2% missing), but there was no evidence of social patterning. Variables collected in later sweeps are affected by the dropout rate of children between GUS survey sweeps [15].

**FIGURE 2:**
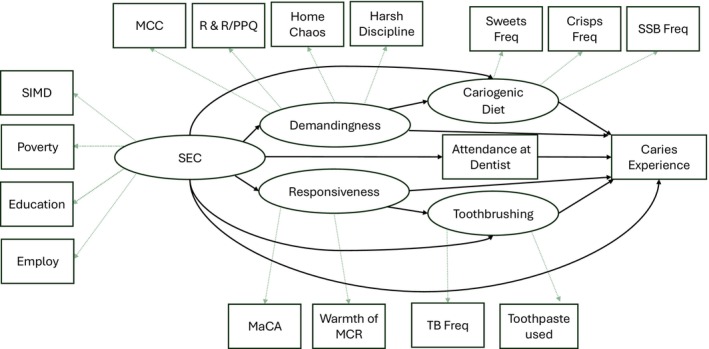
Original structural equation model with observed variables. Toothbrushing is observed, not a latent variable for BC2. Employ, employment level; Freq, frequency; MaCA, mother and child activities; MCC, Mother Child Conflict (Pianta Scale); MCR, Mother Child Relationship (Pianta Scale); R & R/PPQ, Rules and Routines (BC1)/Parenting Practices Questionnaire Authoritarian Score (BC2); SEC, socioeconomic circumstances; SIMD, Scottish Index of Multiple Deprivation; SSB, sugar sweetened beverages; TB, toothbrushing. Circular nodes: Latent variables; Rectangular nodes: Observed variable.

Table 1 summarises cohort characteristics of participants at the first sweep of GUS BC1 or BC2. Table S1 presents caries prevalence stratified by key predictors at the sweep when those variables were collected. All numbers are calculated using GUS survey response weights (Approx BC1: *n* = 3113 and BC2: *n* = 4004). These numbers differ from final SEM models counts shown in Figure 2 due to complete case analysis.

**TABLE 1:** Birth cohorts characteristics.

Variable	Birth cohort 1	Birth cohort 2
Age (years) at National Dental Inspection Programme Exam		
*n*	3171	4769
Min	4.11	4.57
Q1	5.31	5.25
Median	5.54	5.49
Q3	5.80	5.76
Max	6.80	6.99
Sex		
Male	1608 (50.7)	2427 (50.9)
Female	1564 (49.3)	2342 (49.1)
Caries Experience at Age 5		
Yes	954 (30.1)	1330 (27.9)
No	2217 (69.9)	3439 (72.1)

### 3.1 Preliminary Analysis Results

After running the univariable modified Poisson regression models, the theoretical constructs were further specified by incorporating the observed variables, as illustrated in Figure 2. This specification represented the original SEM, which was subsequently refined through an iterative process of testing and modification (S1).

### 3.2 Structural Equation Modelling Results

Measurement models for parenting styles indicated that only one indicator per construct met the loading threshold (≥ 0.4): mother–child activities for parental responsiveness, and home chaos for parental demandingness. As no other indicators loaded strongly onto these constructs, parenting styles were treated as observed variables in the final model to avoid overparameterization.

**FIGURE 3:**
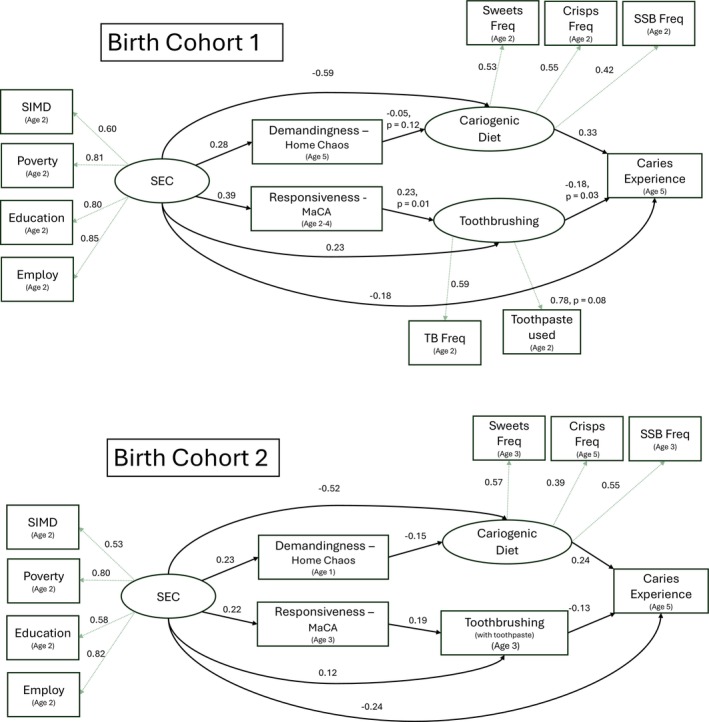
Final models with standardised path coefficients (*p* < 0.01 unless stated). Employ, employment level; Freq, frequency; MaCA, mother and child activities; SEC, socioeconomic circumstances; SIMD, Scottish Index of Multiple Deprivation; SSB, sugar sweetened beverages; TB, toothbrushing. Circular nodes: Latent variables; Rectangular nodes: Observed variables.

The final pathway models diagram examining to what extent parenting styles and oral health behaviours mediate the relationship between SEC and childhood caries experience is presented in Figure 3. All significant pathways were consistent across cohorts. All *p* < 0.01 unless stated.

SEC exhibited direct effects on caries experience (BC1: *β* = −0.18; BC2: *β* = −0.24) as well as indirect effects through parenting and health behaviours. SEC was directly associated with parental responsiveness (BC1: *β* = 0.39; BC2: *β* = 0.22), which in turn was positively associated with toothbrushing (BC1: *β* = 0.23; BC2: *β* = 0.19), though SEC retained an additional direct effect on toothbrushing (BC1: *β* = 0.23, BC2: *β* = 0.12), suggesting partial mediation.

Similarly, SEC was directly associated with higher demandingness (BC1: *β* = 0.28, BC2: *β* = 0.23), which was linked to a lower cariogenic diet (BC1: *β* = −0.05, BC2: *β* = −0.15). Again, SEC had an additional direct effect on the cariogenic diet (BC1: *β* = −0.59, BC2: *β* = −0.52). While parenting styles had no direct effects on caries experience, they exerted small indirect influences through oral health behaviours; responsiveness on toothbrushing and demandingness on diet. Diet showed stronger associations with caries (BC1: *β* = 0.33, BC2: *β* = 0.24) than toothbrushing (BC1: *β* = −0.18, BC2: *β* = −0.13), with effects generally larger in birth cohort 1 (pre‐Childsmile).

Notably, total mediated effects declined from BC1 (58.8%) to BC2 (39.1%), primarily due to reduced direct oral health behaviours mediation (Toothbrushing: 9.5% to 4.0% and Diet: 44.6% to 31.7%), while parenting pathways mediated ≤ 5% of effects in both cohorts (Table 2). Sensitivity analyses confirmed these patterns were robust across alternative model specifications, including analyses using continuous parenting variables and models with dental attendance included.

**TABLE 2:** Mediation percentages of Socioeconomic Circumstances‐Caries Experience pathways in final models.

Pathway	Mediation percentage	
	Birth cohort 1	Birth cohort 2
Demandingness—Cariogenic Diet	1.06	2.10
Responsiveness—Toothbrushing	3.70	1.38
Cariogenic Diet	44.6	31.67
Toothbrushing	9.48	3.96
Total	58.80	39.11

SEM demonstrated excellent fit using standard thresholds for both cohorts (CFI/TLI > 0.95, RMSEA < 0.06, SRMR < 0.08):

- BC1: CFI = 0.988, TFI = 0.983, RMSEA = 0.028 (90% CI: 0–0.049), SRMR = 0.032.
- BC2: CFI = 0.979, TLI = 0.968, RMSEA = 0.033 (90% CI: 0.022–0.043), SRMR = 0.026.

### 3.3 Sensitivity Analysis

Sensitivity analyses supported the stability of the primary model findings. Several sensitivity checks were conducted to assess the robustness of the models. Reversing the order of parenting styles and oral health behaviours led to poorer model fit and reduced interpretability, supporting the original theorised sequence. Including parental stress as an additional mediator did not improve model fit and was therefore excluded. Using variables measured at different time points across cohorts produced minimal changes in estimates, and consistency in measurement timing was prioritised.

## 4 Discussion

This study examined how SEC affect childhood caries experience in Scotland, showing the salient repercussions of these inequalities. SEC exhibit sustained detrimental associations with oral health behaviours and childhood caries experience, as well as enduring relationships with parenting styles. Even after accounting for behavioural and parenting pathways, a substantial proportion of the SEC and caries experience association remained unexplained, with minimal parenting styles mediation.

Despite Childsmile's national implementation in 2011 and the population child oral health improvement observed since, SEC‐caries pathways remained unchanged across cohorts. This persistence may reflect the entrenched nature of SEC effects, which are resistant to short‐term interventions. Broader factors including austerity measures following the 2008 financial crisis, rising childhood povert and food insecurity likely further constrained Childsmile's ability to reduce inequalities, which have remained stubbornly persistent [25, 26]. Six years may also have been too short a period for longstanding patterns in inequalities to shift or for national policies to become fully embedded and generate measurable impact.

Turning to specific behaviours, regular home toothbrushing emerged as a protective factor and showed a consistent positive association with SEC, in conjunction with previous findings [27]. However, its mediating role in caries inequalities declined in the later cohort, possibly due to Childsmile's supervised toothbrushing in nurseries and schools and distribution of free toothbrushing packs, both previously shown to be delivering population‐level caries prevention regardless of socioeconomic background [1, 2].

Cariogenic diet's associations remained relatively stable across cohorts and were less influenced by Childsmile's dietary interventions—including advice from dental practices and health support workers, which have shown thus far to have limited impact [1]. Children from lower socioeconomic groups consistently face barriers to healthy diets, including food availability and affordability, issues rooted in environmental and resource constraints rather than individual behaviour [28]. These reflect systemic challenges that behaviour‐change programmes alone cannot address. Initiatives like the UK‐wide Healthy Start scheme (2006) [29] and Scotland's Best Start Foods (2019) [30] have aimed to reduce these material barriers through food vouchers and prepaid cards, showing some improvement in access for disadvantaged families.

Parenting styles had small indirect effects on caries through oral health behaviours. Responsive parenting was associated with more frequent toothbrushing, while demandingness was modestly associated with a less cariogenic diet. Toothbrushing may be supported through cooperative parent–child interactions and gentle coaxing, creating a feedback loop in which positive interactions encourage consistency, whereas dietary choices may more commonly require structure, rules, limit‐setting and firm guidance. Although diet has also been shown to benefit from higher levels of responsiveness in other studies [31, 32]. Despite this, parenting styles' overall mediating influence was minimal. Their small magnitude may partly reflect measurement limitations, as parenting constructs were represented with single indicators rather than latent variables due to weak factor loadings—an approach known to attenuate path coefficients and potentially underestimate mediation effects [33]. More fundamentally, the overall influence of parenting was overshadowed by the direct and indirect effects of SEC. This finding aligns with the Family Stress Model [34], wherein material constraints increase parental stress and limit resources, constraining the capacity of even positive parenting to offset structural disadvantage. Consequently, while parenting interventions may support specific healthy behaviours, they are unlikely to meaningfully reduce oral health inequalities without concurrent strategies to address underlying economic hardship.

Evidence consistently shows that oral health behaviours alone do not fully account for inequalities. This has been confirmed through structural equation modelling analysis [35], including with similarly aged children [36]. Education can influence oral health behaviours [36], while employment status and income can shape habits like toothbrushing, but behavioural improvements alone rarely eliminate inequalities, underscoring the need for structural solutions.

Despite incorporating parenting factors, a persistent preponderance of SEC remains unexplained by the models, highlighting a potential limitation of behaviour‐focused interventions. This is a matter of paramount importance to Scotland's Childsmile programme. It suggests that broader structural inequalities continue to shape oral health outcomes through mechanisms not fully captured by current approaches. Socioeconomic gradients in childhood caries have been shown to persist even after controlling for known risk factors [3], indicating deeply entrenched effects resistant to behaviour‐ or parenting‐focused interventions alone. Evidence suggests that universal welfare programmes will be more effective than targeted approaches in reducing inequalities and sustaining public support for redistribution. Future research should investigate alternative pathways, further exploring parental stress [37], epigenetic changes [38], wider family/social factors [39], and more detailed neighbourhood‐level deprivation [40].

From a policy perspective, these findings underscore the need to pair clinical and behavioural intervention with broader, equity‐oriented strategies that address the structural determinants of oral health. This could include expanding the community linking role of Childsmile teams with organisations that provide social and economic support. Moreover, while Childsmile combines universal and targeted components, sustained reductions in inequalities will likely require more comprehensive social policies that tackle material deprivation and socioeconomic disadvantage [1]. This includes expanding access to healthy food through schemes like the ‘Healthy Start’ vouchers, embedding oral health into broader poverty‐reduction efforts. Although conducted within the Scottish context, the mechanisms identified are likely relevant to similar high‐income settings with comparable welfare and oral health systems.

### 4.1 Strengths and Limitations

This study draws on two large, prospective, population‐based cohorts in the GUS longitudinal study with further data linkage of routine administrative datasets, enabling robust examination of early‐life pathways linking SEC, parenting, oral health behaviours and clinically assessed childhood caries. The inclusion of two birth cohorts spanning the implementation of a major public health intervention (Childsmile) allows for comparative analysis of structural and behavioural mechanisms over time.

The use of an objective clinical measure of caries is a key strength, reducing reliance on self‐ or parent‐reported outcomes. In addition, the study benefits from high‐quality, multidimensional data on family SECs and parenting, including multiple validated measures. The data also have repeated measures for SECs and some oral health behaviours. The richness of these variables enhances confidence in the measurement of both exposures and mediators, allowing more nuanced pathway analysis than typically possible in oral health research.

The NDIP examination used for the study outcome is slightly limited in detail but provides high population coverage and data quality. Dental caries experience data from basic inspections (presence vs. absence of obvious caries) produced results comparable to detailed assessments on a 20% subsample by trained examiners using British Association for the Study of Community Dentistry criteria.

The use of latent constructs and SEM strengthens the measurement of complex constructs and supports the investigation of indirect pathways. However, the timing and structure of data collection did not allow for a fully temporally ordered longitudinal SEM. As a result, the analysis relied on associations at key timepoints, which limits the ability to establish causal inference between mediators and outcomes. While the hypothesised ordering was supported by theoretical and developmental rationale, alternative model specifications were tested and found to be less consistent with the data.

Furthermore, the use of the WLSMV estimator in Lavaan, while appropriate for categorical data, did not support bootstrapped confidence intervals for indirect effects. Consequently, only point estimates were reported, and uncertainty around mediation pathways could not be fully quantified.

A final possible limitation is that the available survey‐based parenting measures did not support creation of multivariable latent constructs, requiring responsiveness and demandingness to be represented by single indicators. This may limit our ability to capture the multidimensional nature of parenting and may introduce measurement error that could underestimate the mediation effects.

## 5 Conclusion

Despite the population‐level child oral health improvement observed with the national implementation of the Childsmile programme, the underlying pathways linking SEC and childhood caries experience remained largely unchanged across cohorts. Parenting styles exerted minimal influence, while improvements in oral health behaviours—particularly toothbrushing—suggest some positive impact of Childsmile interventions. However, these behavioural shifts were insufficient to eliminate inequalities. The persistent and substantial unexplained effect of SEC highlights the enduring role of structural disadvantage, operating through material, psychosocial and behavioural pathways. To meaningfully reduce oral health inequalities from early life, behaviour‐focused and clinical interventions must be complemented by upstream, equity‐oriented policies that address material deprivation and food insecurity.

## Funding

This work was supported by Borrow Foundation, 31336501.

## Conflicts of Interest

The authors declare no conflicts of interest.

## Supporting information


**Figure S1:** (SF1): Sample derivation flow diagram for the Growing Up in Scotland Birth Cohorts 1 (BC1) and 2 (BC2).
**Table S1:** (ST1): Caries experience prevalence across each variable.
**Table S2:** (ST2): Area under the Curve scores with 95% Confidence Intervals of univariable modified Poisson models against caries experience at age 5 adjusted for age and sex.
**Table S3:** (ST3): Counts of Diet variables not used in final diet latent variable with caries experience prevalence in each exposure level.

## Data Availability

The data that support the findings of this study are available on request from the corresponding author. The data are not publicly available due to privacy or ethical restrictions.
