# Supplementary material for: Pathways Linking Socioeconomic Circumstances to Childhood Dental Caries: The Mediating Role of Parenting and Oral Health Behaviours Before and After Childsmile Implementation
Source: Community Dent Oral Epidemiol. 2026 Mar 9;54(4):462–70. doi: 10.1111/cdoe.70062 (PMC13428041; doi:10.1111/cdoe.70062)
Supplement: Supplementary file 1 — Figure S1: (SF1): Sample derivation flow diagram for the Growing Up in Scotland Birth Cohorts 1 (BC1) and 2 (BC2). Table S1: (ST1): Caries experience prevalence across each variable. Table S2: (ST2): Area under the Curve scores with 95% Confidence Intervals of univariable modified Poisson models against caries experience at age 5 adjusted for age and sex. Table S3: (ST3): Counts of Diet variables not used in final diet latent variable with caries experience prevalence in each exposure level. [file CDOE-54-462-s001.docx]

**Supplementary Materials**

**S1 – SEM Model-Building Algorithm**

**Step 1: Preliminary Analysis**

- Conduct univariable modified Poisson regressions of each candidate variable on caries experience, adjusting for NDIP exam age and sex.
- Evaluate statistical significance (p < 0.05) and predictive performance (AUC).
- Retain variables based on significance and theoretical relevance.

**Step 2: Weighting**

- Apply survey weights developed via model-based techniques to adjust for non-response and sampling design throughout the analysis.

**Step 3: Measurement Model Specification**

Define latent variables for:

- Socioeconomic Circumstances (SEC) using household income poverty, area-based deprivation, parental employment, and education level.
- Parenting Dimensions (Responsiveness and Demandingness) from Pianta scales, parenting practices questionnaire, rules and routines, home chaos, and harsh discipline indicators.
- Cariogenic Diet (latent class variable) from sweet, sugar-sweetened beverage, and crisp consumption variables.
- Toothbrushing (latent for BC1; observed for BC2) based on toothbrushing frequency and toothpaste use.
- Conduct confirmatory factor analysis, retaining indicators with standardized loadings ≥ 0.4 (with justified exceptions).
- Add covariances between residuals of observed variables only if theoretically justified.

**Step 4: Structural Model Specification**

Specify the theoretical model where:

- SEC predicts Parenting Dimensions (Responsiveness and Demandingness).
- Parenting Dimensions predict oral health behaviours: Toothbrushing, Regular Attendance at Dentist, Diet.
- Oral health behaviours predict Caries Experience at age 5.

Include direct paths from SEC to oral health behaviours and Caries Experience to allow for partial mediation.

**Step 5: Model Estimation**

- Estimate the model using WLSMV estimator in the Lavaan package in R, appropriate for categorical and ordinal data.
- Use robust standard errors and Wald z-statistics for p-values to adjust for non-normality.

**Step 6: Iterative Model Refinement**

- Examine path coefficients and p-values.
- Sequentially remove non-significant paths (p > 0.05), retaining borderline paths (0.05 ≤ p ≤ 0.15) if theoretically justified.
- Review modification indices and add covariances between residuals of observed variables only when theoretically defensible.
- Re-estimate the model after each change until final model fit criteria are met.

**Step 7: Model Fit Evaluation**

Confirm final model fit using:

- Chi-square statistic (χ²).
- Comparative Fit Index (CFI) > 0.95.
- Root Mean Square Error of Approximation (RMSEA) < 0.06.
- Standardized Root Mean Square Residual (SRMR) < 0.08.

**Step 8: Mediation Analysis**

- Calculate indirect effects as the product of path coefficients from the independent variable (SEC) to mediators (parenting and behaviours) and from mediators to Caries Experience.
- Compute total effects as the sum of direct and indirect effects.
- Calculate proportion mediated as the ratio of indirect to total effects.
- Note: Bootstrapped confidence intervals for indirect effects are unavailable under WLSMV; hence only point estimates are reported.

**S2 – Variable Description**

***Socioeconomic Circumstances (latent variable)***

***Timing: Age 2 (BC1), Age 3 (BC2)***

- **Household Income Poverty**: Defined by the respondent’s household annual income after tax but before housing costs being less than 60% of median equivalised income in Scotland, using the modified Organisation for Economic Co-operation and Development equivalence scale.^1,2^
- **Area-based deprivation**: Measured using a mix of the Scottish Index of Multiple Deprivation (SIMD) 2006/2012. It is based on 37/38 indicators in seven domains of current income, employment, housing, health, education, skills and training, geographic access to services and crime. SIMD is calculated at data zone level, enabling small pockets of deprivation to be identified. The data zones are ranked from most deprived (1) to least deprived (6,505) on the overall SIMD index. The data are then recorded in fifths (SIMD1-SIMD5), with SIMD1 being the most 20% deprived areas in the population.^3,4^
- **Highest Employment status of parent/carer**: classified using the National Statistics Socio-economic Classification (NS-SEC). For this study, categories were condensed into five groups, with a sixth category, 'never worked'.^5^
- **Highest Education level of parent/carer**: the maximum education of respondent or partner categorised according to the National Vocational Qualifications (NVQ) and Qualifications and Credit Framework (QCF) levels.^6^ The levels are: No Qualification or Other; GCSEs/Standard Grades/NVQ Level 2 or Below; A Levels/Highers/NVQ Level 3 or Equivalent; HNC, HND, NVQ Level 4 or Equivalent; and Degree/NVQ Level 5 or Equivalent.

***Parenting styles:***

***Theoretical Framework: Baumrind’s Framework.***^7^

***Constructs: Responsiveness and Demandingness***

***Note: Different sweep timings and instruments across BC1 and BC2***

***Responsiveness (latent variable)***

- ***Warmth (Pianta-scale) - Timing: Age 5 (BC1), Age 3 (BC2):*** Seven items from the Pianta scale ^8^, with acceptable reliability (Cronbach's alpha = 0.67). Each item was rated on a scale from 1 ("definitely does not apply") to 5 ("definitely applies"), with "can't say" responses treated as missing. Scores were summed for parents who completed all responsiveness items. The approximately lowest third of parents (low warmth) are compared with those who scored higher (High Warmth).
- ***Mother Child Activities - Timing: Age 2-4 (BC1), Age 3 (BC2):*** A count was made of how many of six activities the mother had engaged in with the child in the past week. These activities included reading books or stories, playing outdoors, painting or drawing, singing nursery rhymes or songs, practicing letters or shape recognition, and using a computer or games console. A mean score was computed for each sweep, and these scores were divided into three categories: low (0 to 3 activities), medium (4 activities), and high (5 or 6 activities). For BC2, the data were collected at age 3, and the question regarding computer use was excluded.

***Demandingness (latent variable)***

- ***Rules and Routines (BC1) - Timing: Age 2 & 5:*** At age 2, caregivers were asked about the regularity of meals, with a response of "always." At age 5, caregivers were asked about regular bedtime and whether the child had to tidy up toys, brush teeth, stay in their room, and turn off the TV or music, using a 4-point scale (always, usually, sometimes, never or almost never). The total number of "always" responses was counted excluding the brush teeth due to collinearity, and the number of rules was categorized into low, medium, or high.^9^
- ***Home Chaos - Timing: Age 5 (BC1) and Age 1 (BC2):*** Uses an abbreviated version of the Confusion, Hubbub, and Order scale.^10^ This scale assesses household disorganization.^11^ Scores were grouped into thirds of the population as low, medium, or high.
- ***Harsh discipline - Timing: Age 2 (BC1) and Age 3 (BC2):*** Binary variable asking whether the parents had ever smacked their child.
- ***Mother-Child Conflict (Pianta-scale) - Timing: Age 5 (BC1) and Age 3 (BC2):*** Using eight/seven items from the Pianta scale ^8^ with items scored on a 4-point scale as for the Pianta warmth items (see above). Cronbach’s alpha indicated good reliability (0.80). Scores were grouped into thirds as low, medium and high conflict.
- ***Authoritarian Questions (Parenting Practices Questionnaire) (BC2) - Timing: Age 5:*** Parenting Practices Questionnaire (PPQ)^12^ was used to assess parenting styles. Authoritarian section was used to represent high demandingness.

Further observed variables were trialled as part of latent variables and proved to be non-significant Parental Supervision (Age 4 (BC1) – Demandingness)^13^ and Early mother infant attachment (Age 10 months (BC1/2 – Responsiveness).^14^ Neither were included in final theoretical model.

**Supplementary Figure 1 (SF1): Sample derivation flow diagram for the Growing Up in Scotland Birth Cohorts 1 (BC1) and 2 (BC2)**

**
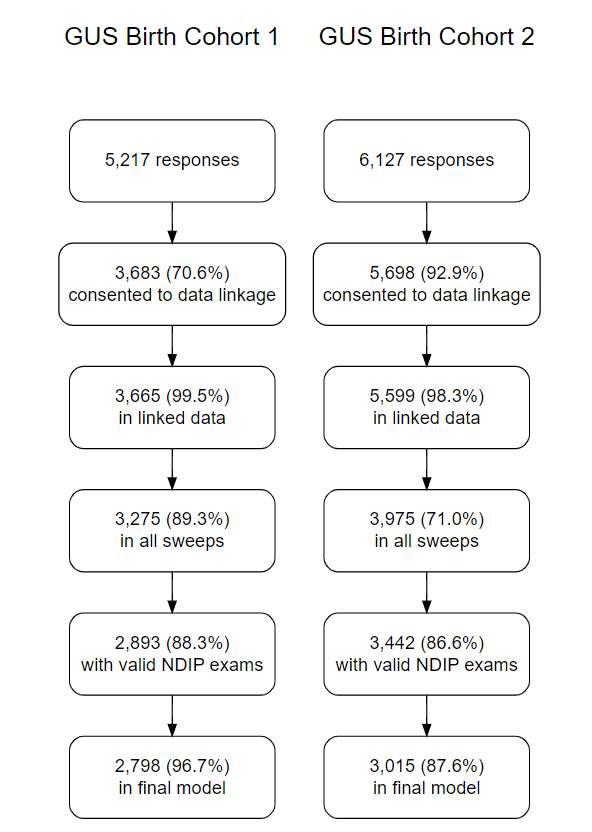
**

**Supplementary Table 1 (ST1): Caries experience prevalence across each variable**

| Age (Cohort 1/Cohort 2) | Variable |  | Birth Cohort 1 | Birth Cohort 2 |
| --- | --- | --- | --- | --- |
|  | Socioeconomic Variables |  | n (%) | n (%) |
| 2/3 | Household Income Poverty | Yes | 385/823 (46.8) | 399/984 (40.5) |
|  |  | No | 531/2140 (24.8) | 526/2527 (20.8) |
|  |  | Missing | 149 | 493 |
|  |  |  |  |  |
| 2/3 | SIMD fifths | 1 | 358/728 (49.2) | 384/948 (40.5) |
|  |  | 2 | 195/565 (34.5) | 262/873 (30) |
|  |  | 3 | 165/586 (28.1) | 199/818 (24.3) |
|  |  | 4 | 142/630 (22.6) | 145/691 (21) |
|  |  | 5 | 102/578 (17.7) | 112/706 (15.9) |
|  |  | Missing | 30 | 0 |
|  |  |  |  |  |
| 2/3 | Household Employment Status | Higher managerial | 363/1613 (22.5) | 335/1914 (17.5) |
|  |  | Intermediate Occupation | 127/465 (27.4) | 149/568 (26.2) |
|  |  | Small employees and own account workers | 68/183 (36.9) | 72/262 (27.5) |
|  |  | Lower supervisory and technical occupations | 108/268 (40.3) | 115/327 (35.2) |
|  |  | Semi routine and routine occupations | 255/519 (49.1) | 370/848 (43.6) |
|  |  | Never worked/long-term unemployed | 49/67 (73.3) | 62/116 (53.4) |
|  |  | Missing | 0 | 4 |
|  |  |  |  |  |
| 2/3 | Household Education Level | Degree/ NVQ level 5 or equivalent | 193/1115 (17.3) | 251/1577 (15.9) |
|  |  | HNC, HND, NVQ level 4 or equivalent | 121/444 (27.2) | 137/550 (24.9) |
|  |  | A levels/Highers/NVQ level 3 or equivalent | 225/633 (35.6) | 251/843 (29.8) |
|  |  | GCSE’s/Standard Grades/NVQ level 2 or below | 316/736 (42.9) | 339/852 (39.8) |
|  |  | No Qualification or Other | 112/181 (61.9) | 115/195 (59) |
|  |  | Missing | 6 | 17 |
|  |  |  |  |  |
|  | Parenting Styles |  |  |  |
| 5/3 | Warmth of Mother Child Relationship | High Warmth (34 – 35) | 521/1870 (27.9) | 554/2251 (24.6) |
|  | (Pianta Scale) | Low Warmth (7 – 33) | 333/970 (34.3) | 459/1560 (29.4) |
|  |  | Missing | 73 | 212 |
|  |  |  |  |  |
| 2-4/3 | Mother Child Activities | High (5 – 6) | 855/2870 (29.8) | 656/2735 (24) |
|  |  | Medium (4) | 97/218 (44.5) | 257/782 (32.9) |
|  |  | Low (0 – 3) | 16/26 (61.8) | 180/489 (36.8) |
|  |  | Missing | 6 | 13 |
|  |  |  |  |  |
| 5 | Rules and Routines | High (5) | 162/564 (28.8) |  |
|  |  | Medium (4) | 595/2089 (28.5) |  |
|  |  | Low (0 – 3) | 127/259 (49.1) |  |
|  |  | Missing | 3 |  |
|  |  |  |  |  |
| 5/1 | Home Chaos | High (10 – 20/9 – 15) | 384/1007 (38.1) | 502/2076 (24.2) |
|  |  | Medium (8 – 9/7 – 8) | 305/1143 (26.7) | 301/1172 (25.7) |
|  |  | Low (4 – 7/3 – 6) | 200/764 (26.2) | 297/783 (37.9) |
|  |  | Missing | 183 | 1 |
|  |  |  |  |  |
| 4/3 | Harsh Discipline | Yes | 167/508 (32.8) | 251/842 (29.8) |
|  |  | No | 799/2604 (30.7) | 830/3158 (26.3) |
|  |  | Missing | 0 | 0 |
|  |  |  |  |  |
| 5/3 | Conflict of Mother Child Relationship | High (19 – 40 /18 – 35) | 841 (33.1) | 1252 (27.7) |
|  | (Pianta Scale) | Medium (13 – 18 /12 – 17) | 1068 (29.3) | 1638 (25.7) |
|  |  | Low (8 – 12 /7 – 11) | 946 (28.8) | 1044 (27.7) |
|  |  | Missing | 57 | 89 |
|  |  |  |  |  |
| 5 | Authoritarian Score | Q1 – Low |  | 187/710 (25.5) |
|  | (Parenting Practices Questionnaire) | Q2 |  | 179/763 (23.5) |
|  |  | Q3 |  | 155/687 (22.6) |
|  |  | Q4 |  | 122/526 (23.2) |
|  |  | Q5 – High |  | 208/699 (29.8) |
|  |  | Missing |  | 54 |
|  |  |  |  |  |
|  | Oral Health Behaviours |  |  |  |
|  | Diet |  |  |  |
| 2/3 | How often child eats sweets? | > 1 (day) | 161/358 (44.8) | 176/461 (38.2) |
|  |  | 1 (day) | 362/982 (36.9) | 532/1705 (31.2) |
|  |  | 5 – 6 (week) | 46/157 (29.6) | 77/291 (26.6) |
|  |  | 2 – 4 (week) | 260/973 (26.7) | 232/1098 (21.1) |
|  |  | 1 (week) | 81/352 (23.1) | 64/318 (20.1) |
|  |  | 1-3 (month) | 20/130 (15.1) | 10/85 (11.6) |
|  |  | Less or never | 39/165 (23.4) | 10/78 (12.9) |
|  |  | Missing | 1 | 0 |
|  |  |  |  |  |
| 2/5 | How often child eats crisps? | > 1 (day) | 162/361 (45) | 29/60 (47.8) |
|  |  | 1 (day) | 371/1052 (35.3) | 203/626 (32.5) |
|  |  | 5 – 6 (week) | 68/250 (27.2) | 68/247 (27.5) |
|  |  | 2 – 4 (week) | 241/879 (27.4) | 303/1277 (23.7) |
|  |  | 1 (week) | 73/306 (23.7) | 122/612 (19.9) |
|  |  | 1-3 (month) | 19/106 (18.2) | 39/248 (15.8) |
|  |  | Less or never | 34/162 (21.3) | 102/369 (27.5) |
|  |  | Missing | 1 | 1 |
|  |  |  |  |  |
| 2/3 | How often child drinks soft drinks? | > 1 (day) | 108/241 (44.8) | 23/36 (62.6) |
|  |  | 1 (day) | 57/128 (44.4) | 29/72 (40.4) |
|  |  | 2 – 6 (week) | 36/82 (44.2) | 49/114 (42.8) |
|  |  | 1 (week) | 42/94 (45.1) | 90/208 (43.3) |
|  |  | 1-3 (month) | 44/94 (46.7) | 78/211 (37) |
|  |  | Less or never | 681/2475 (27.5) | 831/3393 (24.5) |
|  |  | Missing | 1 | 0 |
|  |  |  |  |  |
|  | Toothbrushing |  |  |  |
| 2 | Frequency of toothbrushing | > 1 a day | 643/2266 (28.4) |  |
|  |  | 1 a day | 271/735 (36.9) |  |
|  |  | < 1 a day | 53/114 (46.4) |  |
|  |  | Missing | 0 |  |
|  |  |  |  |  |
| 2 | Toothpaste Used | Yes | 946/3072 (30.8) |  |
|  |  | No | 17/29 (58.9) |  |
|  |  | Missing | 0 |  |
|  |  |  |  |  |
| 3 | Frequency of toothbrushing using fluoride toothpaste | > 1 a day |  | 855/3328 (25.7) |
|  |  | 1 a day |  | 210/647 (32.5) |
|  |  | < 1 a day |  | 35/58 (59.7) |
|  |  | Missing |  | 0 |
|  |  |  |  |  |
|  | Attendance at Dentist |  |  |  |
| 1-5 | Regular Attendance at Dentist (4 out of the first 5 years) | Yes | 299/1250 (23.9) | 416/1859 (22.4) |
|  |  | No | 668/1862 (35.9) | 685/2173 (31.5) |
|  |  | Missing | 0 | 0 |
|  |  |  |  |  |

**Supplementary Table 2 (ST2): Area under the Curve scores with 95% Confidence Intervals of univariable modified Poisson models against caries experience at age 5 adjusted for age and sex**

| Variable | Birth Cohort 1 | Birth Cohort 2 |
| --- | --- | --- |
| Household Income Poverty | 0.609 [0.586, 0.631] | 0.605 [0.583, 0.628] |
| SIMD fifths | 0.648 [0.627, 0.669] | 0.623 [0.602, 0.643] |
| Household Employment Status | 0.629 [0.606, 0.651] | 0.645 [0.625, 0.666] |
| Household Education Level | 0.664 [0.643, 0.685] | 0.652 [0.632, 0.672] |
|  |  |  |
| Parenting Styles |  |  |
| Warmth of Mother Child Relationship | 0.544 [0.521, 0.567] | 0.535 [0.514, 0.556] |
| Mother Child Activities | 0.541 [0.519, 0.563] | 0.543 [0.522, 0.564] |
| Rules and Routines | 0.576 [0.553, 0.599] |  |
| Home Chaos | 0.572 [0.549, 0.595] | 0.558 [0.54, 0.577] |
| Harsh Discipline | 0.528 [0.506, 0.55] | 0.529 [0.508, 0.55] |
| Conflict of Mother Child Relationship | 0.533 [0.51, 0.556] | 0.527 [0.506, 0.548] |
| Authoritarian Score |  | 0.549 [0.527, 0.572] |
|  |  |  |
| Oral Health Behaviours |  |  |
| How often child eats sweets? | 0.611 [0.589, 0.632] | 0.601 [0.582, 0.621] |
| How often child eats crisps? | 0.599 [0.578, 0.621] | 0.590 [0.569, 0.612] |
| How often child drinks soft drinks? | 0.581 [0.559, 0.603] | 0.585 [0.564, 0.606] |
| Frequency of toothbrushing | 0.560 [0.538, 0.583] |  |
| Toothpaste Used | 0.535 [0.513, 0.557] |  |
| Frequency of toothbrushing using fluoride toothpaste |  | 0.551 [0.53, 0.572] |
| Regular Attendance at Dentist (4 out of the first 5 years) | 0.575 [0.553, 0.596] | 0.575 [0.556, 0.593] |

**Supplementary Table 3 (ST3): Counts of Diet variables not used in final diet latent variable with caries experience prevalence in each exposure level**

| Age (Cohort 1/ Cohort 2) | Variable |  | Birth Cohort 1 | Birth Cohort 2 |
| --- | --- | --- | --- | --- |
|  |  |  | (% prevalence of caries) | (% prevalence of caries) |
| 10m | Child drinks: |  |  |  |
|  | Water | No |  | 309/831 (37.2) |
|  |  | Yes |  | 1020/3938 (25.9) |
|  | Baby Juice | No |  | 749/3241 (23.1) |
|  |  | Yes |  | 580/1529 (37.9) |
|  | Fruit Juice (Diet) | No |  | 925/3570 (25.9) |
|  |  | Yes |  | 404/1199 (33.7) |
|  | Fruit Juice (NOT diet) | No |  | 1240/4460 (27.8) |
|  |  | Yes |  | 90/310 (29) |
|  | Fizzy/Soft Drinks | No |  | 1325/4753 (27.9) |
|  |  | Yes |  | 6/17 (35.3) |
|  | Herbal Drinks | No |  | 1308/4722 (27.7) |
|  |  | Yes |  | 21/47 (43.8) |
|  | Tea | No |  | 1240/4538 (27.3) |
|  |  | Yes |  | 93/231 (40.1) |
|  | Coffee | No |  | 1330/4763 (27.9) |
|  |  | Yes |  | 2/7 (28.6) |
|  | Something Else | No |  | 1320/4731 27.9) |
|  |  | Yes |  | 11/38 (28.9) |
|  | Only Milk or Formula | No |  | 1286/4625 (27.8) |
|  |  | Yes |  | 45/144 (31) |
|  |  | Missing |  | 0 |
|  |  |  |  |  |
| 2/3 | How many fruit in a day? | 0 | 40/92 (43.5) | 147/403 (36.5) |
|  |  | 1 | 154/360 (42.8) | 165/610 (27) |
|  |  | 2-3 | 545/1842 (29.6) | 617/2268 (27.2) |
|  |  | 4-5 | 188/686 (27.4) | 151/665 (22.7) |
|  |  | 6+ | 41/135 (30.4) | 14/50 (28) |
|  |  | Missing | 1 | 36 |
|  |  |  |  |  |
|  |  |  |  |  |
| 5 | How many fruit in a day? | 0 | 156/416 (37.5) | 94/359 (26.2) |
|  |  | 1 | 224/646 (34.7) | 221/729 (30.3) |
|  |  | 2-3 | 495/1597 (31) | 540/2086 (25.9) |
|  |  | 4-5 | 71/311 (22.8) | 1043/422 (24.4) |
|  |  | 6+ | 4/18 (21.1) | 7/37 (18.4) |
|  |  | Missing | 12 | 15 |
|  |  |  |  |  |
| 2/3 | How many veg in a day? | 0 | 71/184 (38.6) | 294/917 (32) |
|  |  | 1 | 278/737 (37.7) | 262/915 (28.6) |
|  |  | 2-3 | 537/1899 (28.3) | 425/1793 (23.7) |
|  |  | 4-5 | 69/253 (27.3) | 103/338 (30.4) |
|  |  | 6+ | 10/39 (25.6) | 5/30 (16.1) |
|  |  | Missing | 2 | 34 |
|  |  |  |  |  |
| 5 | How many veg in a day? | 0 | 314/832 (37.7) | 281/915 (30.7) |
|  |  | 1 | 202/644 (31.3) | 199/682 (29.1) |
|  |  | 2-3 | 382/1257 (30.4) | 389/1577 (24.7) |
|  |  | 4-5 | 46/222 (20.6) | 78/379 (20.6) |
|  |  | 6+ | 6/23 (26.1) | 7/56 (12.3) |
|  |  | Missing | 20 | 37 |
|  |  |  |  |  |
| 5 | How often child eats sweets? | 1+ (day) | 145/283 (51.4) | 105/252 (41.7) |
|  |  | 1 (day) | 417/1190 (35) | 446/1472 (30.3) |
|  |  | 5-6 (week) | 69/254 (27.1) | 68/320 (21.2) |
|  |  | 2-4 (week) | 245/954 (25.7) | 263/1132 (23.2) |
|  |  | 1 (week) | 50/221 (22.6) | 48/293 (16.4) |
|  |  | 1-3 (month) | 14/49 (28) | 14/64 (21.9) |
|  |  | Less often or never | 19/48 (39.6) | 27/115 (23.3) |
|  |  | Missing | 0 | 1 |
|  |  |  |  |  |
| 5 | How often child eats crisps? | 1+ (day) | 67/107 (62.6) |  |
|  |  | 1 (day) | 249/609 (40.9) |  |
|  |  | 5-6 (week) | 62/194 (31.8) |  |
|  |  | 2-4 (week) | 339/1132 (29.9) |  |
|  |  | 1 (week) | 121/510 (23.7) |  |
|  |  | 1-3 (month) | 55/243 (22.6) |  |
|  |  | Less often or never | 61/204 (29.9) |  |
|  |  | Missing | 0 |  |
|  |  |  |  |  |
| 5 | How often child has soft drinks? | 1+ (day) | 342/867 (39.4) | 284/827 (34.3) |
|  |  | 1 (day) | 113/386 (29.2) | 101/386 (26.1) |
|  |  | 2-6 (week) | 92/283 (32.5) | 92/347 (26.5) |
|  |  | 1 (week) | 43/148 (28.9) | 62/224 (27.7) |
|  |  | 1-3 (month) | 35/111 (31.5) | 38/158 (24.1) |
|  |  | Less often or never | 329/1203 (27.3) | 394/1707 (23.1) |
|  |  | Missing | 1 | 1 |
|  |  |  |  |  |
| 3 | How often child has fruit juice/smoothie? | 1+ (day) |  | 151/547 (27.6) |
|  |  | 1 (day) |  | 275/991 (27.7) |
|  |  | 2-6 (week) |  | 233/858 (27.2) |
|  |  | 1 (week) |  | 103/389 (26.4) |
|  |  | 1-3 (month) |  | 62/237 (26.2) |
|  |  | Less often or never |  | 278/1013 (27.5) |
|  |  | Missing |  | 0 |
|  |  |  |  |  |
| 3 | How often child has diluting juice/sport drink? | 1+ (day) |  | 672/2031 (33.1) |
|  |  | 1 (day) |  | 177/700 (25.3) |
|  |  | 2-6 (week) |  | 97/408 (23.7) |
|  |  | 1 (week) |  | 19/147 (12.9) |
|  |  | 1-3 (month) |  | 17/104 (16.2) |
|  |  | Less often or never |  | 120/643 (18.6) |
|  |  | Missing |  | 0 |
|  |  |  |  |  |
| 5 | How often child has diet soft drinks? | 1+ (day) | 192/553 (34.7) |  |
|  |  | 1 (day) | 81/225 (36) |  |
|  |  | 2-6 (week) | 83/224 (36.9) |  |
|  |  | 1 (week) | 70/194 (35.9) |  |
|  |  | 1-3 (month) | 35/130 (26.7) |  |
|  |  | Less often or never | 493/1672 (29.5) |  |
|  |  | Missing | 0 |  |
|  |  |  |  |  |
| 5 | How often child has milk? | 1+ (day) | 319/1131 (28.2) |  |
|  |  | 1 (day) | 312/931 (33.5) |  |
|  |  | 2-6 (week) | 137/393 (34.8) |  |
|  |  | 1 (week) | 41/126 (32.5) |  |
|  |  | 1-3 (month) | 11/33 (33.3) |  |
|  |  | Less often or never | 134/385 (34.7) |  |
|  |  | Missing | 0 |  |
|  |  |  |  |  |
| 5 | How often child has water? | 1+ (day) | 370/1294 (28.6) |  |
|  |  | 1 (day) | 196/627 (31.3) |  |
|  |  | 2-6 (week) | 147/432 (34.1) |  |
|  |  | 1 (week) | 52/155 (33.5) |  |
|  |  | 1-3 (month) | 16/59 (27.1) |  |
|  |  | Less often or never | 173/433 (39.9) |  |
|  |  | Missing | 0 |  |
|  |  |  |  |  |
| 3 | Savoury Snack: | No | 565/1641 (34.4) |  |
|  |  | Yes | 403/1472 (27.4) |  |
|  | Crisps | No | 534/2008 (26.6) |  |
|  |  | Yes | 433/1105 (39.2) |  |
|  | Cereal | No | 845/2724 (31) |  |
|  |  | Yes | 123/389 (31.5) |  |
|  | Cakes/Biscuits | No | 649/2068 (31.4) |  |
|  |  | Yes | 319/1046 (30.5) |  |
|  | Fruit | No | 295/760 (38.8) |  |
|  |  | Yes | 672/2353 (28.6) |  |
|  | Bread etc | No | 568/1875 (30.3) |  |
|  |  | Yes | 400/1239 (32.3) |  |
|  | Sweets etc | No | 709/2386 (29.7) |  |
|  |  | Yes | 260/727 (35.7) |  |
|  | Other | No | 903/2924 (30.9) |  |
|  |  | Yes | 65/190 (34.2) |  |
|  | Child not hungry between meals | No | 966/3106 (31.1) |  |
|  |  | Yes | 2/8 (25) |  |
|  |  |  |  |  |
| 5 | Snacking on: |  |  |  |
|  | Savoury Snack: | No | 574/1621 (35.4) |  |
|  |  | Yes | 378/1375 (27.5) |  |
|  | Crisps | No | 462/1602 (28.8) |  |
|  |  | Yes | 489/1395 (35.1) |  |
|  | Cereal | No | 794/2555 (31.1) |  |
|  |  | Yes | 158/442 (35.7) |  |
|  | Cakes/Biscuits | No | 565/1755 (32.2) |  |
|  |  | Yes | 387/1241 (31.2) |  |
|  | Fruit | No | 389/962 (40.4) |  |
|  |  | Yes | 563/2034 (27.7) |  |
|  | Bread etc | No | 601/1856 (32.4) |  |
|  |  | Yes | 350/1140 (30.7) |  |
|  | Sweets etc | No | 635/2028 (31.3) |  |
|  |  | Yes | 316/968 (32.6) |  |
|  | Ricecakes | No | 905/2776 (32.6) |  |
|  |  | Yes | 45/220 (20.5) |  |
|  | Yoghurt | No | 267/982 (27.2) |  |
|  |  | Yes | 683/2015 (33.9) |  |
|  | Other | No | 920/2891 (31.8) |  |
|  |  | Yes | 32/105 (30.2) |  |

# Supplementary References

1. Joint Report on Social Inclusion. Directorate-General for Employment and Social Affairs. (2004).

2. Hagenaars AJM, de Vos K, Zaidi MA. *Poverty statistics in the late 1980s – Research based on micro-data*. European Commission Eurostat Publications Office; 1994.

3. Scottish Government. Scottish Index of Multiple Deprivation 2006 Technical Report. 2006;

4. Scottish Index of Multiple Deprivation 2012: A National Statistics Publication for Scotland: Executive Summary (2012).

5. Pevalin D, Rose D. The National Statistics Socio-Economic Classification: Unifying Official and Sociological Approaches to the Conceptualization and Measurement of Social Class in the United Kingdom. *Sociétés contemporaines*. 2002;45-46(1):75-106. doi:10.3917/soco.045.0075

6. Raffe D. 'Simplicity Itself': The creation of the Scottish Credit and Qualifications Framework. *Journal of Education and Work*. 2003/09/01 2003;16(3):239-257. doi:10.1080/1363908032000099421

7. Baumrind D. Effects of authoritative parental control on child behavior. *Child development*. 1966:887-907.

8. Pianta RC. Child-parent relationship scale. 1992;

9. Growing Up In Scotland: Parenting and children’s health (2011).

10. Coldwell J, Pike A, Dunn J. Household chaos--links with parenting and child behaviour. *J Child Psychol Psychiatry*. Nov 2006;47(11):1116-22. doi:10.1111/j.1469-7610.2006.01655.x

11. Matheny AP, Wachs TD, Ludwig JL, Phillips K. Bringing order out of chaos: Psychometric characteristics of the confusion, hubbub, and order scale. *Journal of Applied Developmental Psychology*. 1995/07/01/ 1995;16(3):429-444. doi:<https://doi.org/10.1016/0193-3973(95)90028-4>

12. Robinson C, Mandleco B, Roper S, Hart C. Authoritative, Authoritarian, and Permissive Parenting Practices: Development of a New Measure. *Psychological Reports*. 12/01 1995;77doi:10.2466/pr0.1995.77.3.819

13. Morrongiello BA, Corbett M. The Parent Supervision Attributes Profile Questionnaire: a measure of supervision relevant to children's risk of unintentional injury. *Inj Prev*. Feb 2006;12(1):19-23. doi:10.1136/ip.2005.008862

14. Condon JT, Corkindale CJ. The assessment of parent-to-infant attachment: Development of a self-report questionnaire instrument. *Journal of Reproductive and Infant Psychology*. 1998/02/01 1998;16(1):57-76. doi:10.1080/02646839808404558

15. Conway DI, McMahon AD, Watling C, et al. National Dental Inspection Programme (NDIP) 2022. 2022;
